# Driving Principle and Stability Analysis of Vertical Comb-Drive Actuator for Scanning Micromirrors

**DOI:** 10.3390/mi15020226

**Published:** 2024-01-31

**Authors:** Yameng Shan, Lei Qian, Junduo Wang, Kewei Wang, Peng Zhou, Wenchao Li, Wenjiang Shen

**Affiliations:** 1 School of Nano-Tech and Nano-Bionics, University of Science and Technology of China, Hefei 230026, China; ymshan2018@sinano.ac.cn (Y.S.);; 2 Suzhou Institute of Nano-Tech and Nano-Bionics, Chinese Academy of Sciences, Suzhou 215123, China

**Keywords:** MEMS micromirrors, stability analysis, electrostatic actuators, self-alignment

## Abstract

We have developed a manufacturing process for micromirrors based on microelectromechanical systems (MEMS) technology. The process involves designing an electrostatic vertically comb-driven actuator and utilizing a self-alignment process to produce a height difference between the movable comb structure and the fixed comb structure of the micromirror. To improve the stability of the micromirror, we propose four instability models in micromirror operation with the quasi-static driving principle and structure of the micromirror considered, which can provide a basic guarantee for the performance of vertical comb actuators. This analysis pinpoints factors leading to instability, including the left and right gap of the movable comb, the torsion beams of the micromirror, and the comb-to-beams distance. Ultimately, the voltages at which device failure occurs can be determined. We successfully fabricated a one-dimensional micromirror featuring a 0.8 mm mirror diameter and a 30 μm device layer thickness. The height difference between the movable and fixed comb structures was 10 μm. The micromirror was able to achieve a static mechanical angle of 2.25° with 60 V@DC. Stable operation was observed at voltages below 60 V, in close agreement with the theoretical calculations and simulations. At the driving voltage of 80 V, we observed the longitudinal displacement movement of the comb fingers. Furthermore, at a voltage of 129 V, comb adhesion occurred, resulting in device failure. This failure voltage corresponds to the lateral torsional failure voltage.

## 1. Introduction

MEMS micromirrors are lightweight, small-size, and cost-effective, making them ideal for a wide range of applications in optical communications [1], autonomous driving [2], and augmented reality (AR)/virtual reality (VR) [3]. Comb-drive micromirrors in particular present notable benefits, including a susceptibility to easy oscillations, rapid response times, and a facilitated precise control of the mirror deflection angle [4,5,6]. They are highly appealing for optical applications, serving purposes in projection displays [7,8], spectrometers [9], optical switches [10], and other applications requiring precise optical control and adjustments [11]. These applications typically demand highly precise microfabrication technology to ensure optical performance and stability [12,13,14].

The driving force of the vertical comb-drive actuator is oriented vertically, effectively mitigating the occurrence of the pull-in effect commonly observed in parallel plate movements [15,16,17]. In an ideal setting, maintaining equal spacing between movable and fixed comb fingers results in mutually canceling lateral forces, establishing a steady state. However, the lateral forces may escalate at high voltages due to alignment accuracy and etching process error factors. In the presence of subtle disturbances, an imbalance in the lateral forces on the left and right sides of the vertical comb-drive structure can manifest, potentially leading to instability and contact between the movable and fixed comb fingers [18]. This contact triggers lateral adhesion, significantly impeding performance and resulting in device failure.

The research on the “pull-in” instability of MEMS devices began as early as the 1960s. Nathanson et al. reported an early analysis in which they used a mass–spring system to simulate the electrostatic deflection of parallel plate actuators, studying the impact of pull-in voltage on resonators [19]. With the widespread application of MEMS devices, the importance of the instability of device “pull-in” is increasingly growing. Hirano et al. conducted a modeling study on the lateral instability of in-plane comb-drive actuators driven by combs [20]. Bochobza-Degani et al. introduced the “pull-in” voltage and critical angle in out-of-plane torsional actuators using polynomial algebraic equations [21]. Pamidighantam et al. investigated the instability of electrostatically driven comb teeth based on the linear beam theory [22]. Zhou and Dowd analyzed the side instability of comb-drive actuators using a one-dimensional model with structural lateral translational stiffness [23]. Huang and Lu established a two-dimensional model to examine the influence of lateral and angular offsets on the critical voltage of in-plane comb-drive MEMS actuators [24]. Lee and Solgaard came up with three failure modes of the scanners, and analytical expressions of pull-in deflection were obtained by applying 2D analytical capacitance models to the derived pull-in equations [25].

In this work, a thorough analysis is conducted to refine the conditions leading to instability in the vertical comb-drive micromirror. Various scenarios are examined for a more comprehensive understanding of the underlying instability factors. This paper introduces the principle of quasi-static deflection for an electrostatic micromirror with a vertical comb-driven structure and provides a detailed explanation of the forces generated by the unequal-height comb-finger structure. Four potential instability models that may manifest during the operation of the micromirror are explored in connection with its structure. One model involves comb fingers with a relatively large length-to-width ratio, causing an uneven force distribution and resulting in the elastic deformation of the comb fingers themselves. The other three models involve comb fingers with high rigidity that concentrate the force on the twist beams, inducing a bending deformation of the fixed elastic beam at both ends. Consequently, the movable comb finger changes position and comes into contact with the fixed comb finger, causing the device to stop functioning. Failure voltages are determined based on these models. These models establish a robust foundation to support the stable operation of electrostatic micromirrors at high voltages, effectively compensating for the accuracy errors introduced in the fabrication process and ensuring that the mirror operates in a steady state. A test system is set up to validate the analysis of the vertical comb-driven micromirror device. This system ultimately determines the stable operating voltage for the device.

## 2. The Principle and Design of Electrostatically Actuated Micromirrors

### 2.1. Analysis of Drive Structure and Quasi-Static Deflection Principle

**Figure 1:**
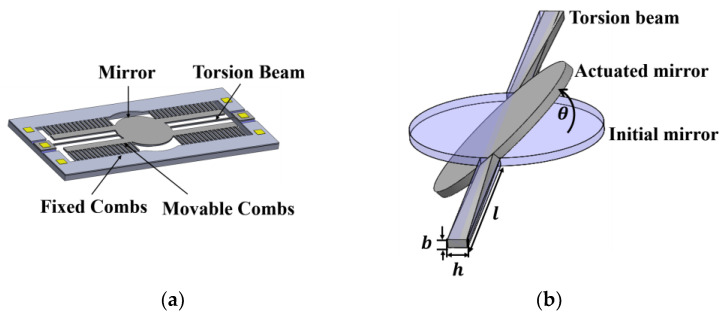
(**a**) Schematic diagram of electrostatic vertical comb-drive micromirror; (**b**) simplified model of one-dimensional micromirror torsion.

Designing a MEMS micromirror involves careful consideration of torsion beams, mirror size, and the structure of the micromirror comb [26]. For the vertical comb-drive micromirror, its primary operational mode is characterized by static deflection. Figure 1a illustrates an electrostatically actuated scanning micromirror with rectangular torsion beams. Four sets of comb-driven structures are connected to the sides of the circular mirror. It consists of two rows of undersized combs, one of which is attached to the substrate as the fixed comb, while the other is attached to the mirror as the movable comb.

According to the definition of torsion for noncircular cross-section beams in mechanics of materials, for a straight bar with a rectangular cross section undergoing free torsion, the calculation formula for the relative rotation angle θ between the ends of the beam with applied torque T is as follows:

(1) $$\theta =\frac{Tl}{GI_{t}}$$

In the equation, $GI_{t}$ is referred to as the torsional stiffness of the beam:

(2) $$GI_{t}=G\beta hb^{3}$$

(3) $$G=\frac{E}{2\left(1+\mu \right)}$$

where h represents the length of the long side of the torsion beam’s cross section, b represents the length of the short side of the torsion beam’s cross section. l is the length of the torsion beam, as shown in Figure 1b. E is Young’s modulus of silicon material; μ is the Poisson’s ratio. The factor β is related to the torsion of the rectangular cross-section beam and is dependent on the ratio of the side lengths h/b. Factor β for rectangular cross-section beam torsion is listed in Table 1.

**Figure 2:**
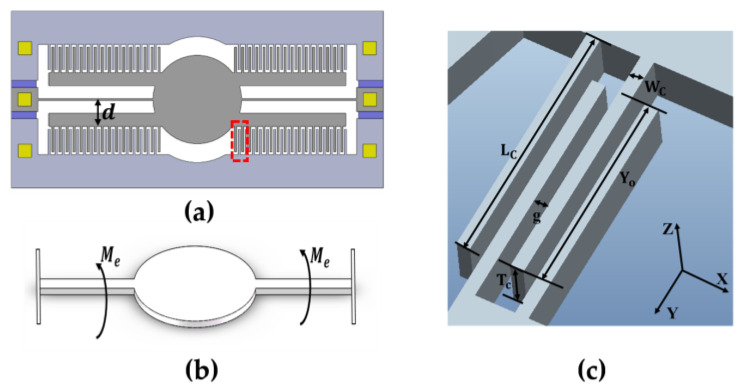
(**a**) The 1D schematic of an electrostatic comb-drive micromirror, including the comb-finger structure, mirror surface, and torsion beams; (**b**) the micromirror subjected to an external torque on one side; (**c**) the detailed schematic of the micromirror’s comb-finger structure (The red box part in subfigure (**a**)), highlighting the specific structural parameters of the movable and fixed comb finger.

The external torque for driving the torsional motion of the micromirror is provided by the vertical comb-drive structure. A change in the electric field between the comb-drive structures results in the generation of a force that induces the deflection of the micromirror. In the quasi-static torsional mode, the system attains a static equilibrium state wherein the torque experienced by the torsional beam T is the applied torque. $M_{e}$ represents the external torque exerted by the combs.

(4) $$T=M_{e}=F_{Z}d$$

where $F_{Z}$ is the electrostatic force between the combs in the vertical direction, and d is the distance from the torsion beam to the end of the movable comb finger. As shown in Figure 2, the single-beam micromirror undergoes torsional motion due to the driving force provided by the comb-finger structure in the Z-direction.

The electric energy $W\left(\theta \right)$ between the interdigitated comb electrodes can be approximated as the energy stored in a capacitor [28]. The expression for this energy is:

(5) $$W\left(\theta \right)=\frac{1}{2}C\left(\theta \right)V^{2}$$

A movable comb finger is affected by the fixed comb finger on the left and right sides, therefore, the total electric energy generated by comb fingers become:

(6) $$W_{e}\left(\theta \right)=NC\left(\theta \right)V^{2}$$

where N is the number of comb fingers, V is the driving voltage, and $C\left(\theta \right)$ is the capacitance between the movable comb finger and the fixed comb finger. The electrostatic force between the comb fingers can be calculated using the derivative of the total electric energy with respect to the rotation angle, which is given by:

(7) $$F_{Z}\left(\theta \right)=\frac{\partial W_{e}\left(\theta \right)}{\partial \theta }=N\frac{\partial C\left(\theta \right)}{\partial \theta }V^{2}$$

where $\frac{\partial C\left(\theta \right)}{\partial \theta }$ is related to the rate of change in the overlap area between the movable and fixed comb fingers. The capacitance formula between the plate electrodes is given by:

(8) $$C\left(\theta \right)=\frac{\varepsilon }{g}A\left(\theta \right);\frac{\partial C\left(\theta \right)}{\partial \theta }=\frac{\varepsilon }{g}\frac{\partial A\left(\theta \right)}{\partial \theta }$$

where ε is the permittivity of the air, $A\left(\theta \right)$ represents the overlapping area between the movable and fixed comb fingers, and g is the gap distance between the movable and fixed comb finger.

**Figure 3:**
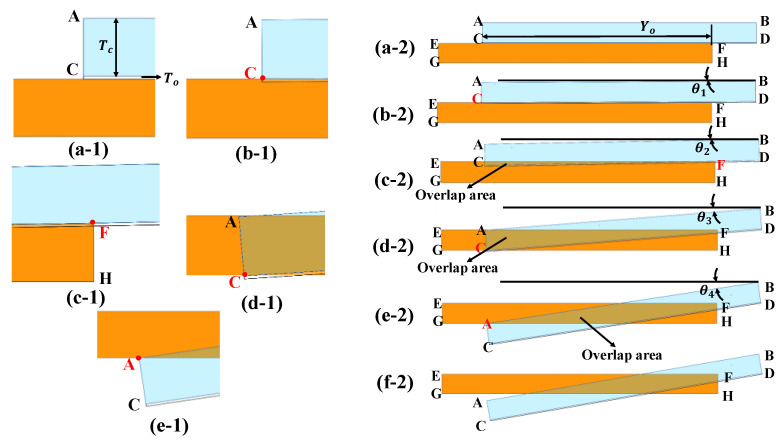
Changes in the overlap area of the comb finger. When the comb finger rotates around the central flexure beam with angle θ, the overlap area of the upper (fixed) and lower (movable) comb fingers changes. (**a-1**,**a-2**) are the initial state diagrams of the comb finger; (**b-1**,**b-2**) when the deflection angle is $\theta_{1}$, point C intersects the upper edge of the fixed comb finger, and the overlap area is zero; (**c-1**,**c-2**) when the deflection angle is $\theta_{2}$, point F intersects with the upper edge of the fixed comb finger, and the overlap area is a triangle; (**d-1**,**d-2**) when the deflection angle is $\theta_{3}$, point C intersects the lower edge of the fixed comb finger, and the overlap area is a quadrilateral; (**e-1**,**e-2**) when the deflection angle is $\theta_{4}$, point A intersects the lower edge of the fixed comb finger, and the overlap area is an irregular pentagon; (**f-2**)when point A intersects with the lower edge of the fixed comb finger, the overlap area becomes smaller.

When a voltage is applied between the movable and fixed comb fingers, electrostatic forces are generated in the vertical direction. The rotation angle between the comb fingers is considered to be θ in this particular state. As the torsion beam of the micromirror twists, the overlap area between the movable and fixed comb fingers undergoes irregular changes [29,30,31], which can be categorized into the following situations, where the fringing field effects are small. As shown in Figure 3(a-1), in the presence of a dielectric layer with a thickness of $T_{o}$ between the movable and fixed comb fingers, the dielectric layer acts as an insulating material, preventing direct contact between the comb fingers and creating a gap between them. Figure 3(a-2) shows the status of the comb fingers.

Case 1 is shown in Figure 3(b-1). Point C on the left side of the movable comb finger is in contact with the upper edge of the fixed comb finger, and the deflection angle $\theta_{1}$ is given in Figure 3(b-2). There is no overlap area between the movable and fixed comb fingers, so the overlap area is zero.

The maximum angle of torsion is denoted by $\theta_{1}$:

(9) $$\theta_{1}=\tan^{-1}\left(\frac{T_{o}}{d+Lc}\right)$$

where d is the distance from the torsion beam to the end of the movable comb finger. $L_{c}$ is the length of the comb finger. When $0<\theta <\theta_{1}$, the resulting overlap area of a single finger gap is:

(10) $$A\left(\theta \right)=0$$

The incremental area change in the single finger gap is:

(11) $$\frac{\partial A\left(\theta \right)}{\partial \theta }=0$$

Case 2 is shown in Figure 3(c-1). The lower edge of the movable comb finger touches the right peak F of the fixed comb finger. As the angle continues to increase, the overlap area forms a triangle, and Figure 3(c-2) shows that the maximum angle at this time is $\theta_{2}$.

The maximum angle of torsion is denoted by $\theta_{2}$:

(12) $$\theta_{2}=\tan^{-1}\left(\frac{T_{o}}{d+Lc-Y_{o}}\right)$$

where $Y_{o}$ is the overlap length of the movable and fixed comb fingers. When $\theta_{1}<\theta <\theta_{2}$, the resulting overlap area of a single finger gap is:

(13) $$A\left(\theta \right)=\frac{1}{2}\frac{{\left[\left(d+Lc\right)\tan\theta -T_{o}\right]}^{2}}{\tan\theta }$$

The incremental area change in the single finger gap is:

(14) $$\frac{\partial A\left(\theta \right)}{\partial \theta }=\frac{1}{2}\left[{\left(\frac{\left(d+Lc\right)}{\cos\theta }\right)}^{2}-{\left(\frac{T_{o}}{\sin\theta }\right)}^{2}\right]$$

Case 3 is shown in Figure 3(d-1). When the movable comb finger’s point C intersects the lower edge of the fixed comb finger, the maximum angle is $\theta_{3}$. As the angle continues to increase, the overlap area forms a quadrilateral, as shown in Figure 3(d-2).

The maximum angle of torsion is denoted by $\theta_{3}$:

(15) $$\theta_{3}=\tan^{-1}\left(\frac{T_{o}+T_{c}}{d+Lc}\right)$$

where $T_{c}$ is the thickness of the comb finger. When $\theta_{2}<\theta <\theta_{3}$, the resulting overlap area of a single finger gap is:

(16) $$A\left(\theta \right)=\frac{1}{2}{\left(L_{C}+d\right)}^{2}\tan\theta -\left(L_{C}+d\right)T_{o}-\frac{1}{2}{\left(d+Lc-Y_{o}\right)}^{2}\cos\theta \sin\theta \;+\left(d+Lc-Y_{o}\right)\left(\cos\theta \right)T_{o}$$

The incremental area change in the single finger gap is:

(17) $$\frac{\partial A\left(\theta \right)}{\partial \theta }=\frac{1}{2}\left[{\left(L_{C}+d\right)}^{2}\sec^{2}\left(\theta \right)+{\left(d+Lc-Y_{o}\right)}^{2}\left(\sin^{2}\left(\theta \right)-\cos^{2}\left(\theta \right)\right)-2(d+Lc\;-Y_{o})T_{o}(\sin\theta )\right]$$

Case 4 is shown in Figure 3(e-1). As the torsion angle continues to increase, the shape becomes an irregular pentagon before the movable comb finger’s point A intersects the lower edge of the fixed comb finger. Figure 3(e-2) shows the diagram of the maximum angle $\theta_{4}$ of this process.

The maximum angle of torsion is denoted by $\theta_{4}$:

(18) $$\theta_{4}=\sin^{-1}\left(\frac{T_{o}+T_{c}}{d+Lc-T_{o}}\right)$$

When $\theta_{3}<\theta <\theta_{4}$, the resulting overlap area of a single finger gap is:

(19) $$A\left(\theta \right)=\frac{1}{2}{\left(L_{C}+d\right)}^{2}\tan\theta -\left(L_{C}+d\right)T_{o}-\frac{1}{2}{\left(d+Lc-Y_{o}\right)}^{2}\cos\theta \sin\theta \;+(d+Lc-Y_{0})(\cos\theta )T_{o}-\frac{1}{2}Y_{o}^{2}\tan\theta +\frac{1}{2}T_{c}Y_{o}$$

The incremental area change in the single finger gap is:

(20) $$\frac{\partial A\left(\theta \right)}{\partial \theta }=\frac{1}{2}\left[{\left(L_{C}+d\right)}^{2}\sec^{2}\left(\theta \right)+{\left(d+Lc-Y_{o}\right)}^{2}\left(\sin^{2}\left(\theta \right)-\cos^{2}\left(\theta \right)\right)-2(d+Lc\;-Y_{o})T_{o}\sin\theta )\right]-\frac{1}{2}Y_{o}^{2}sec^{2}\left(\theta \right)$$

Case 5 is shown in Figure 3(f-2). As the twisting angle continues to increase, the shape becomes an irregular hexagon, and at this point, the overlap area starts to decrease. The capacitance decreases, and the driving force direction changes. In theory, this situation will not occur. The change in overlap area with the angle is a nonlinear function of the micromirror twisting angle, increasing from the rest position until $\theta =\theta_{4}$ (see Figure 3(f-2)).

Combining Equations (1)–(4) and (7)–(8), we calculate the mirror rotation angle θ:

(21) $$\theta =\frac{Tl}{G\beta hb^{3}}=\frac{\varepsilon NlV^{2}}{G\beta ghb^{3}}\frac{\partial A\left(\theta \right)}{\partial \theta }$$

### 2.2. Analysis of Micromirror Driving Stability

The analysis of the micromirror driving stability involves examining the equilibrium and dynamic behavior of the mirror under different operating conditions. Various factors would influence the stability of the micromirror, such as its mechanical structure, electricity, and control aspects. This section mainly discusses and analyzes the mechanical stability of the micromirror.

**Figure 4:**
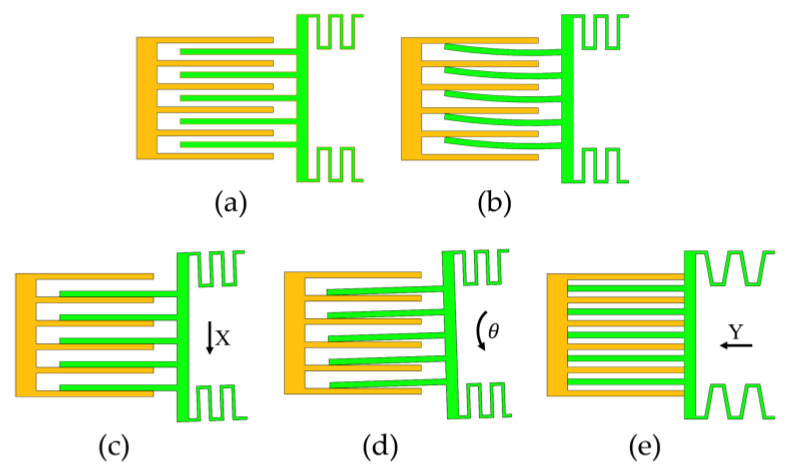
Several scenarios of structural instability. (**a**) In the initial state of the movable and fixed comb fingers, under normal conditions, the comb fingers are in force equilibrium, and the structure remains stable; (**b**) the comb fingers’ lateral bending contact’s instability model; (**c**) the comb fingers’ lateral displacement contact’s instability model; (**d**) the comb fingers’ lateral rotational contact’s instability model; (**e**) the comb fingers’ lengthways displacement contact’s instability model.

The driving force of the micromirror in the vertical comb-drive structure is primarily provided by the electrostatic force between fixed and movable comb fingers. The driving force increases as the gap between the comb fingers decreases. However, due to the residual stress in fabrication or alignment deviation, the micromirror may experience small driving forces in directions other than the desired deflection direction. Besides the force generated in the deflection direction, there may be minor driving forces in other directions. The contact between the movable and fixed comb fingers results in a short-circuit failure of the device [29,30,31]. Several scenarios of structural instability are illustrated in Figure 4 as shown below. These models are established based on the micromirror being deflected to its maximum allowable angle, specifically at the position where the overlap area between the movable and fixed comb fingers is maximized.

#### 2.2.1. The Comb Fingers’ Lateral Bending Contact’s Instability Model

**Figure 5:**
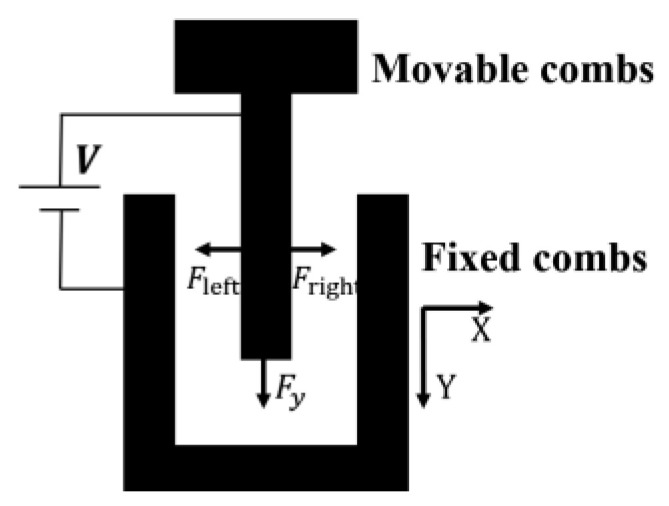
Schematic view of the comb finger’s electrode.

Figure 5 schematically illustrates one movable electrode comb finger placed between fixed electrode comb fingers. In this figure, V is the applied voltage across the electrodes, g is the gap between the fixed and movable comb fingers, $T_{c}$ is the thickness of the upper layer of the movable comb finger, and $Y_{o}$ is the length of the overlap of the comb finger. Assume that the movable electrode finger moves x in the X direction.

(22) $$F_{\mathrm{right}}=\frac{\varepsilon T_{c}Y_{o}}{2{\left(g-x\right)}^{2}}V^{2};F_{\mathrm{left}}=\frac{\varepsilon T_{c}Y_{o}}{2{\left(g+x\right)}^{2}}V^{2}$$

where $F_{\mathrm{right}}$ and $F_{\mathrm{left}}$ represent the lateral force on the right side and left side, respectively, and ε is the permittivity of free space.

In this case, the lateral forces on both sides of the comb finger are no longer balanced, and the electrostatic force $F_{x}$ generated by both sides of the parallel plate is:

(23) $$F_{x}=F_{\mathrm{left}}-F_{\mathrm{right}}=\frac{\varepsilon T_{c}Y_{o}}{2{\left(g-x\right)}^{2}}V^{2}-\frac{\varepsilon T_{c}Y_{o}}{2{\left(g+x\right)}^{2}}V^{2}$$

where $T_{c}$ is the thickness of the movable comb fingers. A positive value of $F_{x}$ proves that the suspended comb is unstable. It looks as if there was a “negative” spring. The equivalent “negative” spring constant $k_{e}$ when the movable comb finger is placed at the center of the gap is

(24) $$k_{e}=\frac{\partial F_{x}}{\partial x}=\frac{\varepsilon T_{c}Y_{o}}{{\left(g-x\right)}^{3}}V^{2}+\frac{\varepsilon T_{c}Y_{o}}{{\left(g+x\right)}^{3}}V^{2}$$

In this case, the spring constant of the movable comb finger bending along the X direction is given by:

(25) $$k_{xc}=\frac{{ET_{c}W_{c}}^{3}}{{12L_{c}}^{3}}$$

where E is Young’s modulus, and $W_{c}$ and $L_{c}$ are the width and length of the comb finger, respectively.

The mechanical spring with spring constant $k_{xc}$ keeps the position of the movable comb finger against the instability of the electrostatic force.

If

(26) $$\left|k_{e}\right|>\left|k_{xc}\right|$$

the movable comb finger stays stable; otherwise, as shown in Figure 4b, the movable comb finger become unstable and touches the fixed comb fingers. The voltage at this point is referred to as the lateral side-instability voltage. At this stage, the electrostatic force between the comb finger undergoes a rapid change, resulting in the attraction between them. Hence, to keep the stable operation of the comb structure [32], Equation (26) must be satisfied.

When the driving voltage exceeds the so-called side-instability voltage $V_{1}$, the comb drive becomes unstable leading to a side-sticking of the movable and fixed comb fingers. By combining Equations (23)–(26) the voltage at which the side instability occurs can be expressed as:

(27) $$V_{1}=\sqrt{\frac{{EW_{c}}^{3}{\left(g-x\right)}^{3}+{\left(g+x\right)}^{3}}{{12\varepsilon L_{c}}^{3}Y_{o}}}$$

#### 2.2.2. The Comb Fingers’ Lateral Displacement Contact’s Instability Model

**Figure 6:**
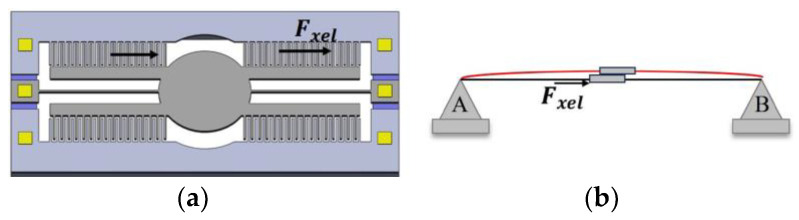
The comb fingers’ lateral displacement contact’s instability model. (**a**) The driving of one side of the comb-finger structure results in a total force in the X direction; (**b**) a schematic diagram showing the deformation of the beam anchored at both ends due to axial forces.

When the elastic constant of the comb finger is large enough, it is difficult to generate the bending contact of the comb finger, and the generated force is transferred to the torsion beam through the root of the comb finger. As shown in Figure 6, the torsion beam anchored at both ends experiences axial forces (also known as tension or compression forces), resulting in axial expansion and contraction deformation. The left beam of the mirror extends due to tensile forces, while the right side undergoes compression forces, causing it to shorten, as illustrated in Figure 4c. As a result, lateral displacement occurs when the movable comb fingers make contact with the fixed comb fingers, creating variations in electrical potential that disrupt the circuit, leading to device instability and an eventual cessation of operation.

The total force of the comb structure in the X direction is:

(28) $$F_{xel}=\frac{N\varepsilon T_{c}Y_{o}}{2{\left(g-x\right)}^{2}}V^{2}-\frac{N\varepsilon T_{c}Y_{o}}{2{\left(g+x\right)}^{2}}V^{2}$$

where n is the number of movable comb finger.

The elastic constant of the torsion beam in the X direction is:

(29) $$k_{xl}=\frac{2ET_{l}W_{l}}{l}$$

The analysis process is similar to that of the previous case. The equivalent “negative” spring constant $k_{e2}$ when the torsion beam bends up or down is:

(30) $$k_{e2}=\frac{\partial F_{xel}}{\partial x}=\frac{N\varepsilon T_{c}Y_{o}}{{\left(g-x\right)}^{3}}V^{2}+\frac{N\varepsilon T_{c}Y_{o}}{{\left(g+x\right)}^{3}}V^{2}$$

It must satisfy $\left|k_{e2}\right|>\left|k_{xl}\right|$. Finally, the instability voltage $V_{2}$ in this case is obtained by

(31) $$V_{2}=\sqrt{\frac{2EW_{l}\left[{\left(g-x\right)}^{3}+{\left(g+x\right)}^{3}\right]}{n\varepsilon lT_{c}Y_{o}}}$$

#### 2.2.3. The Comb Fingers’ Lateral Rotational Contact’s Instability Model

**Figure 7:**
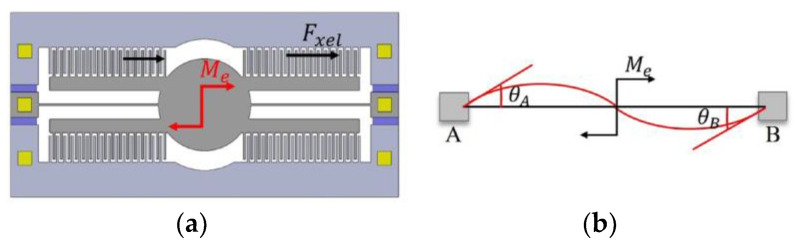
The comb fingers’ lateral rotational contact’s instability model. (**a**) Driving one side of the comb-finger structure generates a total force in the X direction; (**b**) a simplified diagram illustrating the bending deformation of the beam supported and anchored at both ends due to an external torque.

According to the Figure 7a, the lateral electrostatic force $F_{xel}$ generated between the comb finger creates a torque on the torsion beam. In this case, the motion model of the beam is as shown in Figure 7b, where the distortion of the torsion beam causes deflection in the comb finger connected to the beam, resulting in contact between the movable and fixed comb fingers, leading to device instability. Meanwhile, the deflection angle of the movable comb finger is the same as that of the midpoint of the torsion beam.

$\theta_{A}$ and $\theta_{B}$ represent the angular displacements of the cross sections at points A and B along the torsional beam, respectively. The deflection angle of the midpoint of the torsion beam can be calculated based on the applied torque and the torsional stiffness of the system:

(32) $$\theta_{o}=\frac{\theta_{A}}{2}=\frac{\theta_{B}}{2}=\frac{M_{e}2l}{12EI}=\frac{F_{xel}dl}{6EI}$$

where I is the moment of inertia, which is a measure of an object’s resistance to changes in its rotational motion. It quantifies how mass is distributed around the beam of rotation. In the context of a torsion beam, the inertia moment refers to the moment of inertia of the beam itself, which depends on its geometry and mass distribution:

(33) $$I=\frac{l\cdot W_{l}^{3}}{12}$$

The movable comb-finger structure is fixed on the mirror through connecting beams, so the movement of the comb fingers follow the deflection of the mirror structure. In other words, it experiences an angular displacement at the midpoint of the torsional beam. The angular displacement at the midpoint of the torsional beam represents the deflection angle θ of the movable comb fingers:

(34) $$\theta =\tan\left(\frac{g-x}{d+L_{c}}\right)$$

When θ≥ $\theta_{o}$, the movable comb finger touches the fixed comb finger and results the device fails. In this case, the voltage is referred to as the rotational pull-in voltage $V_{3}$:

(35) $$V_{3}=\sqrt{\frac{2\cdot \tan^{-1}\left(\frac{g-x}{d+L_{c}}\right)\cdot E\cdot l\cdot W_{l}^{3}}{n\varepsilon T_{c}Y_{o}\cdot \left[\frac{1}{{\left(g-x\right)}^{2}}-\frac{1}{{\left(g+x\right)}^{2}}\right]\cdot d\cdot 2L}}$$

#### 2.2.4. The Comb Fingers’ Longitudinal Displacement Contact’s Instability Model

In this model, when a voltage is applied to the movable and fixed combs, the overlap area in the direction of the comb overlaps increases, resulting in the generation of electrostatic forces in the Y direction:

(36) $$F_{y}=\frac{N\varepsilon T_{c}}{2g}V^{2}$$

**Figure 8:**
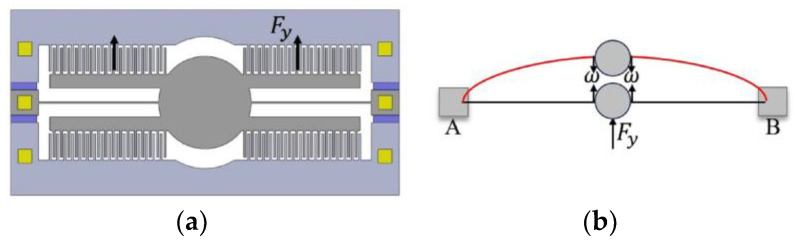
The comb fingers’ longitudinal displacement contact’s instability model. (**a**) Driving one side of the comb-finger structure generates a total force in the Y direction; (**b**) a simplified diagram of the deformation at the midpoint of an elastic beam fixed at both ends under the influence of a longitudinal force; ω represents the maximum deflection.

When the electrostatic force acts on the torsion beam through a mirror, the flexural model of the torsion beam is depicted in Figure 8b. According to the deformation formula for beams under simple loading in materials mechanics, with the ends of the beam securely fixed and subjected to a longitudinal force, the maximum deflection of the beam occurs at its midpoint:

(37) $$\omega =\frac{F_{y}\cdot {\left(2l\right)}^{3}}{48EI}$$

The displacement ($\Delta y=L_{c}-Y_{o}$) required for the device to fail after the contact between the movable and fixed comb fingers is established in the Y direction.

When $\omega \geq L_{c}-Y_{o}$, the contact between the movable comb-finger and the fixed comb-finger structure leads to the failure of the device.

By combining Equations (36) and (37), we obtain the longitudinal unstable voltage at that moment:

(38) $$V_{4}=\sqrt{\frac{L_{c}-Y_{o}ET_{l}W_{l}^{3}g}{l^{3}N\varepsilon T_{c}}}$$

According to the above analysis, the structural parameters of the designed micromirror are determined. The geometric parameters and material properties [28] used in the MEMS fabrication of the micromirror are listed in Table 2.

**Figure 9:**
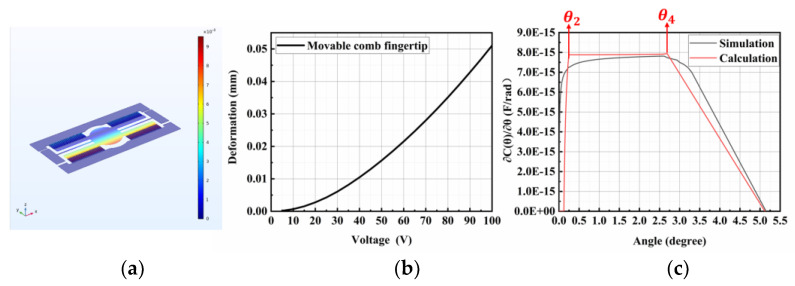
(**a**) The simulation result of the Z-direction displacement of the micromirror. (**b**) The displacement of the movable comb fingertip in the Z direction. (**c**) Incremental capacitance change $\frac{\partial C\left(\theta \right)}{\partial \theta }$ using finite element simulation and theoretical calculation for a limited angular range.

When the given values are substituted into Equations (8)–(20), it is possible to calculate the micromirror incremental capacitance change ($\frac{\partial C\left(\theta \right)}{\partial \theta }$) versus the torsional angle under different driving voltages. To validate the structural design, a three-dimensional model was created based on the parameters mentioned above, and a finite element analysis was conducted using specialized software [33,34]. Finite element analysis (FEA) software is a tool used in engineering to simulate and analyze how structures behave under different conditions. It helps designers optimize designs by predicting factors like stress and deformation. We performed simulations via the finite element method (FEM). Figure 9a illustrates the simulation results of the structural displacement in the Z direction, Figure 9b shows the Z-direction displacement of the tips of the movable comb fingers, and Figure 9c demonstrates the incremental change in capacitance between the comb fingers. The device operated within the range of $0<\theta <\theta_{4}$. Due to the idealized nature of theoretical calculations, where edge effects and other factors are not considered, the calculated values were slightly greater than the simulated values.

## 3. Fabrication

**Figure 10:**
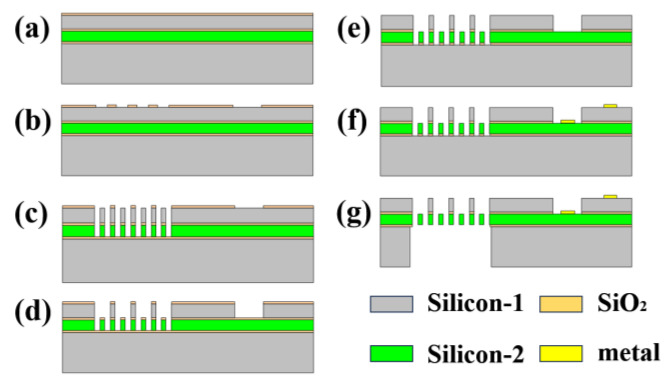
Simplified fabrication process flow for vertical comb drive with self-aligned gaps. A single lithography step defines the gap between the upper and lower fingers. Steps (**a**–**g**) show cross sections during micromirror fabrication processing.

The fabrication process involved bonding two silicon-on-insulator (SOI) wafers together using a silicon-to-silicon wafer bonding technique. Subsequently, a substrate silicon layer was etched away, resulting in the formation of two silicon conducting layers. The silicon dioxide of the top layer was used as a mask, using a single photolithography step and a self-aligning process that created a height difference between the movable comb structure and the fixed comb structure of the micromirror. The fabrication process steps of the vertical comb-drive structure of the electrostatic micromirror are depicted in Figure 10. A multilayer SOI wafer was bonded together (Figure 10a). The first step involved photolithography on the top silicon dioxide layer to create a pattern, followed by dry etching to remove the oxide layer, acting as a mask for the third step to remove the top silicon layer as shown in Figure 10b. The second step encompassed a pattern formation and multiple stages of dry etching. This process included etching two oxide layers and two silicon layers sequentially to produce comb structures of equal height, as illustrated in Figure 10c. After removing the photoresist and cleaning, Figure 10d shows the deep silicon etching of the entire structure to form the unequal-height comb structures. The oxide layer structure was etched using a hydrofluoric acid (HF or BOE) solution to expose the second device layer (Figure 10e). The third step involved a front-side sputtering of metal material and using a wet-etching process to form electrodes (Figure 10f). Subsequently, a temporary bonding process was employed to etch the backside cavity, fully exposing the device layer and reducing the device mass to increase the deflection space (Figure 10g). The final step involved chip cleaning using a wet-cleaning process.

**Figure 11:**
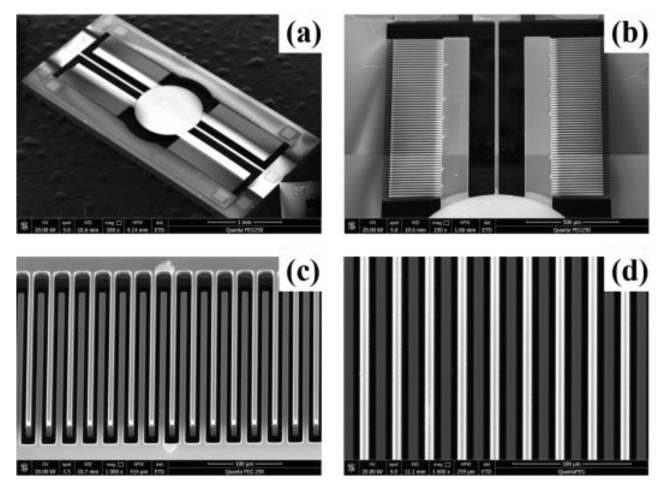
SEMs of fabricated MEMS micromirror: (**a**) whole device; (**b**) micromirror torsion beam and vertical comb-finger structure distribution; SEMs of self-aligning vertical comb fingers: (**c**) 3D view and (**d**) top view.

The vertical comb-drive micromirror devices were fabricated and used to verify the correctness of the above model. Figure 11a shows the overall SEM image of the MEMS micromirror, with a mirror diameter of 0.8 mm. Figure 11b shows a symmetrical arrangement of vertical comb-finger structures on both sides of the torsion beam. This structure allowed the mirror to deflect at a certain angle in both left and right directions. A closed-up view of the vertical comb structure is shown in Figure 11c. In these images, the upper structure of the movable comb fingers is about 10 μm above the lower structure of the fixed comb fingers. Figure 11d shows the gaps on both sides of the movable comb fingers are symmetrical. The upper and lower comb fingers also aligned well.

## 4. Experiments and Discussion

**Figure 12:**
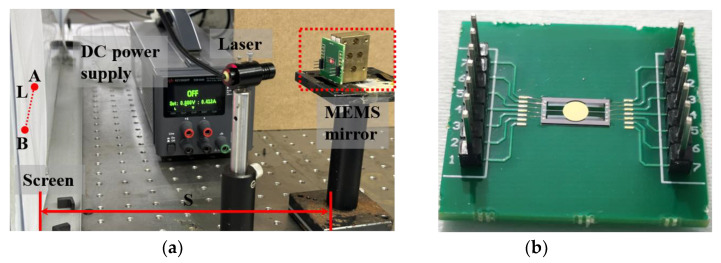
(**a**) Schematic of the optical measurement setup. L represents the length of the light ray on the screen, S is the vertical distance from the mirror to the screen. (**b**) Photo of an assembled MEMS micromirror.

The rotation angle of the micromirror was determined by measuring the distance traveled by a laser beam reflected from the mirror surface when the micromirror was actuated. The static response of the MEMS mirror was measured using an experimental setup that involved a laser beam incident to the center of the mirror plate. The setup comprised a DC power supply, a laser, a MEMS mirror, and a screen, as shown in Figure 12a. The micromirror chip, after processing the wafer, was securely mounted onto a PCB (Printed Circuit Board) for ease of applying the driving voltage, as illustrated in Figure 12b. The experimental testing process was as follows: Firstly, the micromirror chip was securely fixed onto an optical platform, and a signal was applied using a direct current power source. Next, the laser beam was aligned with the mirror surface. Finally, after the mirror surface was deflected, the point projected onto the screen moved from A to B. By measuring the displacement of the laser point and the distance from the micromirror to the screen, the mechanical rotation angle of the micromirror was calculated.

**Figure 13:**
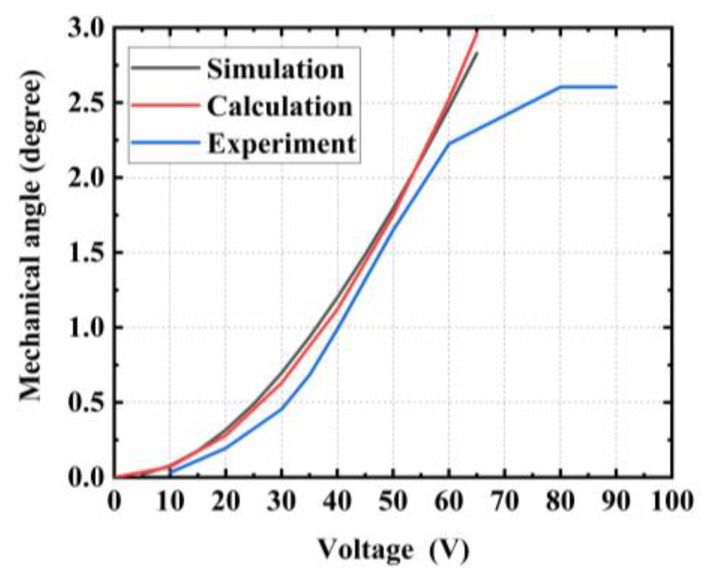
Static response of the MEMS micromirror.

As shown in Figure 13, when the driving voltage increased, the angle of deflection continuously increased. At the driving voltage of 60 V, the deflection angle reached 2.25°. When comparing these experimental results with the simulated and calculated results, it was found that the experimental results were slightly lower. This could be attributed to the fact that in reality, the gap between the comb fingers on the chip was larger than 5 μm, resulting in a smaller driving force than the ideal value, which in turn reduced the deflection angle. Continuing to increase the driving voltage, the angle increased slowly, reaching 2.6° at 80 V. The main reason for this phenomenon is that under a high voltage, the lateral force increases, and under small disturbances, the vertical comb-drive structure is unbalanced, causing the torsion beam to bend and deform, resulting in the actual mechanical deflection angle of the micromirror being less than the ideal value. After further increasing the voltage, the torsion angle of the micromirror remained unchanged. The reason for this phenomenon is that under quasi-static deflection, the torsion angle of the micromirror is affected by the change in the overlap area between the comb fingers. When the angle reached the maximum allowable deviation angle between the comb fingers (2.6°), a further voltage application did not significantly change the angle of the micromirror. At that point, an obvious instability in the lateral rotation and movement of the comb finger was observed. Therefore, the stable working voltage should be less than 80 V.

**Figure 14:**
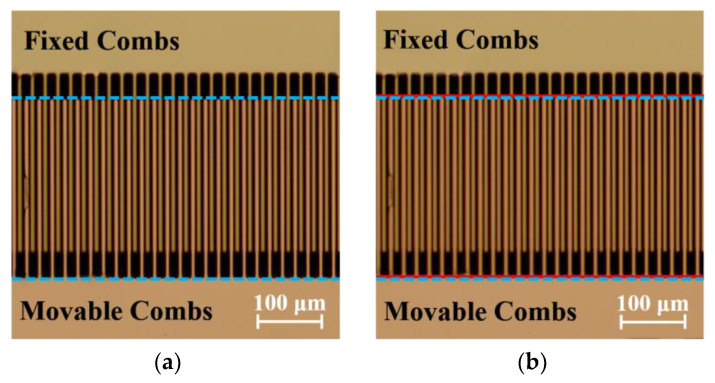
(**a**) The comb finger’s image before applying the test voltage. (**b**) The comb finger’s image after applying the test 80 V voltage. The blue dashed line represents the condition with no applied voltage, while the red solid line represents a slight displacement of the movable comb finger after applying a driving voltage of 80 V.

As the voltage continued to increase, the quasi-static deflection angle of the micromirror remained unchanged. However, the increasing voltage introduced unbalanced forces in other directions on the micromirror’s comb fingers. According to the equilibrium stability analysis, all parameters of the micromirror were substituted into Equations (27), (31), (35), and (38), respectively. The calculated unstable voltages were as follows: $V_{1}=$ 313.12 V, $V_{2}=$ 34,846 V, $V_{3}=$ 129 V, and $V_{4}=$ 237 V. The observation and testing were conducted under an optical microscope. As shown in Figure 14, the partial image of the comb fingers structure was observed. The blue dashed line represents the condition with no applied voltage, while the red solid line represents a slight displacement in the Y direction of the movable comb finger after applying a driving voltage of 80 V. This observation provides a clear explanation for the deviation of the actual deflection angle of the microscope from the theoretical and simulated values during testing within the voltage range of 60 V–80 V. However, since there was no contact between the comb finger, the micromirror continued to operate.

**Figure 15:**
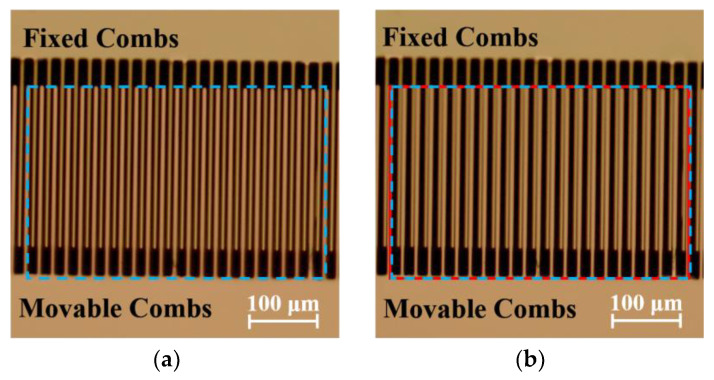
(**a**) The comb finger’s image before applying the test voltage. (**b**) The comb finger’s image after applying the test 129 V voltage. The blue solid line frame represents the comb finger image before testing, while the red dashed line frame represents the comb finger image after testing.

With the continuous increase in voltage, when the voltage reached 129 V, the micromirror underwent a torsional motion in the horizontal direction, causing contact between the movable and fixed comb fingers. At that point, the micromirror became nonfunctional and in the lateral rotational contact instability state. After turning off the voltage, the micromirror returned to its original static state, as shown in Figure 15. The blue solid line frame represents the comb finger image before testing, while the red dashed line frame represents the comb finger image after testing. A comparison reveals that the movable comb finger shifted to the left and contacted with the fixed comb finger. This corresponded to the previously analyzed lateral torsional pull-in voltage values. Through testing, the stable operating voltage range of the micromirror was 0 V–80 V, and the device failed at 129 V. Utilizing this analysis provides a better understanding of the micromirror’s unstable phenomenon and helps to confirm the unstable voltage in the operational mode.

## 5. Conclusions

This paper established the principle of quasi-static deflection for an electrostatic micromirror with a vertical comb-driven structure and provided a detailed explanation of the forces generated by the unequal-height comb-finger structure. Four potential instability models that may manifest during the operation of the micromirror were explored in connection with its structure. The experimental testing effectively validated the analysis of the driving principles and stability model of the vertically comb-driven micromirror. This micromirror was driven by a direct current voltage, achieving a mechanical angle of 2.25° with 60 V@DC. When the driving voltage was below 60 V, stable operation was observed, and it was generally consistent with theoretical calculations and simulations. At a driving voltage of 80 V, the comb-finger structure was observed to undergo a longitudinal movement, and when the driving voltage reached 129 V, a comb-finger adhesion phenomenon occurred, leading to device failure. This failure voltage corresponded to the lateral torsional failure voltage.

In order to make the micromirror torsion angle larger during design, the torsion beam is usually a thin and elongated structure, which is more likely to produce unstable pull-in phenomena and cause device failure. The establishment of the above model can calculate the torsion angle and failure voltage during the design stage, thus greatly reducing the workload. Devices with errors introduced during the manufacturing process can also be analyzed to determine the stable operating voltage so that the micromirror can operate stably. This method can effectively ensure the long-term efficient and stable operation of the device.

## Figures and Tables

**Table 1:** Factor β for rectangular cross-section beam torsion [27].

h/b	1.0	1.2	1.5	2.0	2.5	3.0	4.0	6.0	8.0	10.0	∞
β	0.141	0.166	0.196	0.229	0.249	0.263	0.281	0.299	0.307	0.313	0.333

**Table 2:** Geometric parameters and material properties.

Symbol	Parameters	Value
l	Length of torsion beam	1200 μm
$W_{l}/b$	Width of torsion beam	20 μm
$T_{l}/h$	Thickness of torsion beam	30 μm
d	Distance from the torsion beams to the end of the movable comb fingers	194 μm
g	Gap between fixed and movable fingers	5 μm
$W_{c}$	Width of comb fingers	5 μm
$L_{c}$	Length of comb fingers	280 μm
$T_{c}$	Thickness of movable comb fingers	10 μm
$T_{c2}$	Thickness of fixed comb fingers	20 μm
$Y_{o}$	Length of the overlap of the comb finger	240 μm
$T_{o}$	Thickness of dielectric layer	1 μm
N	Number of movable comb fingers	120
E	Young’s modulus of silicon	130 GPa
*µ*	Poisson’s ratio of silicon	0.28

## Data Availability

The data presented in this study are available on request from the corresponding author.
